# Evaluation of Control Program Against *Streptococcus agalactiae* Infection in Dairy Herds During 2019–2021 in Emilia-Romagna Region, Northern Italy

**DOI:** 10.3389/fvets.2022.904527

**Published:** 2022-06-23

**Authors:** Marco Tamba, Rossella Rocca, Alice Prosperi, Giovanni Pupillo, Patrizia Bassi, Giorgio Galletti, Enrica Martini, Annalisa Santi, Gabriele Casadei, Norma Arrigoni

**Affiliations:** ^1^Department of Emilia-Romagna, Istituto Zooprofilattico Sperimentale della Lombardia e dell'Emilia Romagna, Brescia, Italy; ^2^General Directorate of Personal Care, Health and Welfare, Region of Emilia-Romagna, Bologna, Italy

**Keywords:** cattle, control program, mastitis, Italy, *Streptococcus agalactiae*, Group B streptococcus

## Abstract

*Streptococcus agalactiae* (group B Streptococcus, GBS) is a causative agent of mastitis in dairy cattle, mainly causing a subclinical disease associated with a high somatic cell count (SCC), and a consequent decrease in production yield and quality of milk. GBS has been almost eradicated in many Northern European countries, but there are warnings of its re-emergence as a zoonotic threat. In Italy, only two regions carry out a GBS control program: Lombardy and Emilia-Romagna. In Emilia-Romagna, the program has been in place since 2019 and provides for the bacteriological culture of bulk-tank milk (BTM) of all dairy farms every 6 months and the voluntary application of herd eradication programs in the case of positive results. To assess the progress of the program in Emilia Romagna, in terms of herd-level prevalence and GBS transmission between herds, we analyzed the results of 17,056 BTM cultures from 2,831 dairy herds, sampled bi-annually in the period 2019–2021 (six rounds total). The impact of GBS infection on SCC and milk production was also evaluated. The results show a decreasing trend in both the incidence rate (from 3.0 to 1.5%) and apparent prevalence (from 8.9 to 5.2%) of GBS over the study period. By using a susceptible-infected-susceptible (SIS) model for the estimation of the transmission parameters, a basic reproductive number R_0_ of 1.4 was calculated, indicating an active spread of GBS in the dairy cattle population of the Emilia-Romagna region. GBS infected farms have a consistently higher BTM SCC than negative ones (+77,000 cells/ml), corresponding to a 0.4 kg/cow/day milk loss. Moreover, GBS infected herds resulted in almost three times more likelihood of having non-marketable milk by exceeding the legal SCC limit. This study demonstrates the need to maintain the current control program against GBS to lower its occurrence and prevent significant market losses to farmers.

## Introduction

*Streptococcus agalactiae* (group B Streptococcus, GBS) is a causative agent of mastitis in dairy cattle, with detrimental effects on animal wellbeing and dairy farm profitability. Raboisson et al. (1) estimated a mean loss of €121 (€103–227) per case of clinical mastitis due to *Streptococcus* spp. Although milk loss following clinical mastitis (independent of the pathogen) is well documented (2), pathogen-specific estimates for milk loss and somatic cell count (SCC) due to GBS are currently lacking. In a recent study, the GBS intramammary infection (IMI) was associated with a reduction in milk production yield of 2.5 kg milk/day since 1–2 months before diagnosis and an increase of SCC since 4–5 months before diagnosis (3).

Although clinical cases do occur, GBS predominantly causes chronic and subclinical intramammary infections characterized by high SCC (4) and a reduction in milk production, leading to a substantial economic impact on dairy producers (5). The quality of cheese and other milk products is decreased when cows with subclinical mastitis have a high SCC (6), and a high SCC has a negative effect on milk composition and technological traits (7). Herds infected with GBS usually produce bulk-tank-milk (BTM) with high SCC and total bacteria counts (8), which may limit milk marketing due to non-compliance with the limit of 400,000 cells/ml adopted by the European Union since 1992 (9).

Group B Streptococcus has long been considered a typical contagious pathogen, with the main reservoir localized to the mammary gland of infected cows and transmission occurring during milking (5). However, although not considered one of the “environmental Streptococci,” it is now accepted that GBS can be of environmental origin (10). Some authors showed that GBS could colonize the gastrointestinal tract, and it could be isolated from several environmental sites in affected dairy herds, such as floors, cow beds, and water troughs (11, 12). Multi-locus sequence typing (MLST) of the isolates was performed, and the results suggested that some sequence types (ST) of *S. agalactiae*, such as ST 103, might be more persistent in the environment than others (11).

While GBS often leads to chronic subclinical infections with a low rate of self-cure (5), it is highly sensitive to antibiotics, leading to a high cure rate following treatment (9). GBS has been eradicated from Canada and is near to eradication in some North-European countries, such as Denmark, Norway, and The Netherlands, through control programs developed by the National Mastitis Council and known as the “5-Points plan,” which include: (i) effective post-milking test dipping, (ii) use of antibiotic dry cow therapy in every quarter at the end of each lactation (blanket dry cow therapy, BDCT), (iii) appropriate treatment of clinical cases, (iv) culling of chronically infected cows, and (v) maintenance of milk equipment to ensure stable teat end vacuum (5). To rapidly achieve eradication, systematic identification and treatment of all infected animals (“blitz therapy”) associated with improved hygiene during milking was applied (5, 9). However, BDCT conflicts with the current European legislation, which requires the prudent use of antibiotics (European Parliament and Council Regulation 6/2019) (13).

In recent years, GBS has been considered a reemerging pathogen in some Northern European countries, facing a raising herd-level prevalence, despite the implementation of eradication programs (14–16). The re-occurrence of GBS is probably correlated, in addition to farm management regulation, to the decline of dairy herd number, and the increase of the average herd size. A higher herd size leads to a higher labor demand, which could increase the risk of herd infection since humans may serve as a source of infection for cattle (17, 18). Moreover, a rapid herd expansion may increase the need for purchasing new animals, which may result in an increased risk of introducing GBS (16, 19, 20).

Group B Streptococcus can infect humans, causing different clinical forms, such as bacteremia, skin and soft tissue infections, urinary tract infections, and occasionally necrotizing fasciitis, arthritis, toxic shock syndrome, endocarditis, meningitis, and pneumonia (21–23). The frequent and generally asymptomatic colonization of the gastrointestinal and urogenital tracts (24, 25) of pregnant women is the main cause of infections in newborns during childbirth (21). A recent study carried out in Italy on sympatric GBS isolates, typed by a combination of molecular methods, reported the finding of human and bovine isolates (20.9% out of 203 strains) with common genotypes and antibiotic resistance profiles, supporting the hypothesis of interspecies transmission of GBS between bovines and humans (26), as already reported by other authors (27, 28). More than one strain can be present in a cattle herd, and generally, the cow-adapted strains tend to have a higher within-herd prevalence than shared strains (26, 29).

In Italy, clinical mastitis due to GBS is a notifiable disease, but it is probably underreported, and only two regions (Lombardy and Emilia-Romagna) out of 21 have in place GBS control programs (30, 31). A control plan for GBS was started in the 1980s in dairy herds of the province of Brescia (Lombardy region, Northern Italy), which accounts for about 10% of the national milk production (32). The plan was extended to all the provinces of the Lombardy region in 2012 to reduce the GBS herd-level prevalence below 8% (31). The estimated herd-level prevalence reported in these two regions is about 7–10% (26), while data on GBS occurrence in other Italian regions are not available.

Conversely, GBS infection is not listed in the European Union Animal Health Law (Regulation UE 429/2016) and probably all the sanitary measures against contagious mastitis due to GBS will be repealed in Italy shortly. For these reasons, in 2019, the Emilia-Romagna region, Northern Italy, implemented a control program against GBS, with the following aims: (i) to estimate and reduce the herd-level prevalence of GBS in the region; (ii) to raise the awareness among milk producers about the need of implementing eradication programs in infected herds; (iii) to enhance milk quality and production by reducing the amounts of unmarketed milk due to high SCC or antimicrobial treatment, and (iv) to promote the prudent use of antibiotics in dairy farms through the application of herd eradication programs against contagious mastitis agents, also considering the recent prohibition of prophylactic use of antimicrobials for BDCT since 28 January 2022 (Regulation UE 6/2019). In this study, we used the results of the tests performed on BTM collected in the first 3 years of the GBS surveillance program of the Emilia-Romagna region with the following aims: (i) evaluating the trend of GBS occurrence in the dairy herds of the region; (ii) estimating the GBS transmission rate between herds; and (iii) estimating the impact of the GBS infection on milk production using the BTM SCC.

## Materials and Methods

### Study Population

On 31 December 2021, the National Cattle Database reported 6,290 farms and 573,483 cattle heads in the Emilia-Romagna region, corresponding to 4.6 and 10.2% of the Italian cattle population, respectively (source: https://www.vetinfo.it/j6_statistiche/#/). In the Emilia-Romagna region, most of the cattle are dairy, raised in the freestall barns without the use of pasture. The milk is mainly used for the production of fine cheeses, such as Parmigiano Reggiano and Grana Padano. In the 3 year period, 2019–2021, there has been a progressive decrease in the number of dairy farms, while the number of cows has slightly increased (Table 1). This has led to a progressive increase in the average size of farms from 134.9 to 141.1 cattle per herd. In Italy, health guarantees for animal movements between dairy farms are mandatory for only three diseases: bovine tuberculosis, brucellosis, and enzootic bovine leucosis. Any additional health guarantees are linked to direct agreements between farmers. In this scenario, GBS-infected herds generally have no limits to the movement of cattle between farms.

**Table 1:** Dairy cattle at 31st December. Emilia-Romagna Region 2019–2021.

**Year**	**Number of farms^***a***^**	**Total cattle**	**Number of cows**	**Other cattle**	**Average herd size**	**Percentage difference compared to 2019**		
						**Farms**	**Cattle**	**Cows**
2019	3,591	484,418	234,832	249,586	134.9	Ref.	Ref.	Ref.
2020	3,517	487,539	236,809	250,730	138.6	−2.1%	0.6%	0.8%
2021	3,452	487,104	235,979	251,125	141.1	−3.9%	0.6%	0.5%

*^a^Farms composed by only heifers and dry cows are included in these figures*.

### Milk Samples

Bulk-tank milk samples were collected every 6 months by the Official Veterinary Services in the framework of the Bovine Brucellosis surveillance program, which is mandatory for all dairy farms. The milk was agitated in the bulk tank for 15 min, and then 120 ml of milk, collected from the surface using sterile materials, were transferred into a sterilized plastic container. Samples were immediately transported under refrigeration to the laboratory and maintained at 4 ± 2°C until the analysis.

### Sample Analysis

Bacteriological analysis was performed within 24 h of the collection according to internal standardized procedures adopted by IZSLER. Samples were plated in parallel on the Tallium Kristalviolette Tossin (TKT) agar medium, using two different volumes of inoculum:

a) In the first volume, 100 μl of milk was pipetted into the center of a plate of TKT agar medium and then spread on the surface using a sterilized bent glass rod. A limit of detection of 10 CFU/ml and a sensitivity and a specificity of 99% (each) are attributed to this inoculum (32);
b) In the second volume, 10 μl of milk was spread on a plate of TKT agar medium. A limit of detection of 100 CFU/ml, a sensitivity of 98%, and a specificity of 99% are attributed to this inoculum (32). This inoculum is used to avoid false-negative results due to high bacterial contamination of the bulk milk sample.

Both plates were aerobically incubated at 37°C and examined at 24 and 48 h. Suspect colonies with bluish pigmentation and a β-hemolytic area were submitted to the CAMP test and Gram staining for confirmation. CAMP-positive and Gram-positive colonies were identified as GBS. A sample was considered positive when one or more GBS colonies were identified on at least one of the culture plates.

### Case Definition

A case was defined as a farm from which GBS was cultured from its BTM sample, as previously described. A non-case was a farm whose milk gave a negative result for the pathogen in the same period.

### Data on SCCs

Data, covering the period 2019–2021 on SCC of BTM samples, were collected from Agrishare (http://www.agrishare.com/). Agrishare is a portal, promoted by the Region of Emilia-Romagna, containing integrated databases for various agro-food chains. Joining the portal is voluntary, but over 97% of dairy producers are registered at present.

Farm code, date of sampling, SCC, and SCC geometric moving average were collected. The SCC moving average was calculated on a 90 days period, with at least one sample per month.

### Statistical Analysis

Starting from the model developed by Zadoks et al. (33), we performed the same analyses described in Mweu et al. (16). We calculated the apparent prevalences (APs) and their associated 95% exact binomial confidence intervals (*CI*s) for each semester.

As in open populations, the calculation of rates as opposed to risks is befitting, so we computed the incidence rates (INCr) based on an approximation of the amount of herd-time at risk for the rate denominator as follows:


$$\begin{array}{l} \text{INCr}=\left(\text{Number of newly infected herds in a specific period}\right)/\\ \left(\text{average number at risk}\right)\times \text{Time}, \end{array}$$

where average number at risk = number at risk at the start of the period + [0.5 × (susceptible entries + recoveries – cases – susceptible exits)].

The associated 95% *CI*s were calculated using an exponential error factor (EF) for incidence rates: lower *CI* limit: rate × EF^−1^; upper *CI* limit: rate × EF, where EF = exp (1.96/√d) and d is the rate numerator.

To assess the risk for a GBS positive herd of having BTM exceeding the legal limit for BTM SCC (400,000 cells/ml), we used the prevalence ratio. For each semester and status (positive/negative), the prevalence (P) was calculated as followed:


$$\begin{array}{l} {\text{P}}_{\text{x}}\text{=}(\text{number of herds with status x having at least one BTM }\\ \text{ SCC moving average value }>\text{400},\text{000})\text{/}(\text{number of }\\ \text{ herds with status x having at least one BTM SCC }\\ \text{ moving average value}). \end{array}$$

We used the farm code to link the cultures and BTM SCC datasets.

**Figure 1:**
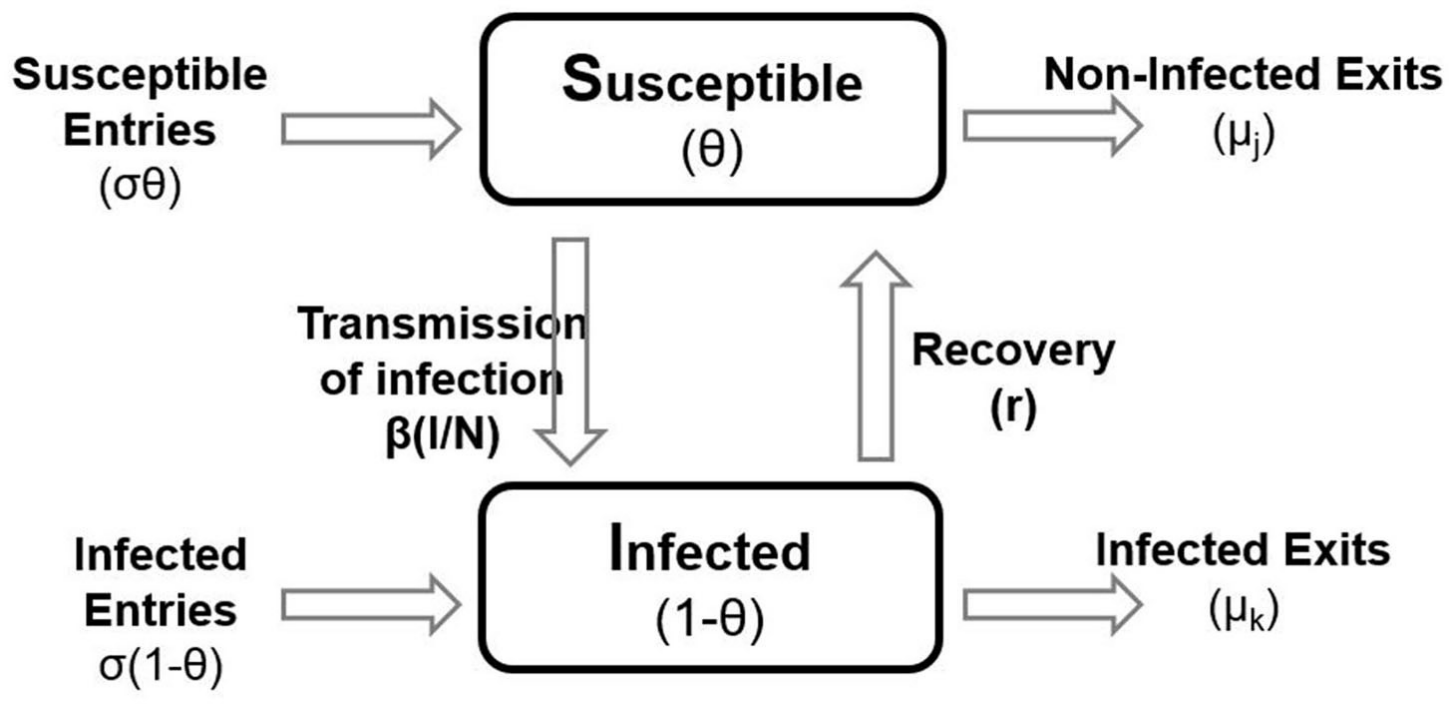
A schematic representation of the susceptible–infected–susceptible model used for the estimation of the transmission parameter, β in the population. The boxes represent the state variables and the arrows represent the flow rates between them. Lettering represents the variables and parameters in the model.

To estimate the transmission characteristics of GBS between herds, we used the framework of a susceptible–infected–susceptible (SIS) model (Figure 1), considering that infected herds upon recovery are capable of being reinfected. Thus, the population of dairy herds was partitioned into Susceptible (S; non-cases) and Infected (I; cases) states, accordingly to the BTM culture result. New herd infections with GBS were assumed to occur at the rate β × S × I/*N*, where β is the transmission rate; S is the number of susceptible herds; I is the number of infected herds, and *N* is the total number of herds present in a specific period (33).

The number of “susceptible entries” and “infected entries” for each 6-month period was calculated by counting as “entry” a herd not present in the previous 6-month period and then classified as “Susceptible” or “Infected” depending on its status. The number of “Non-infection exits” and “Infection exits” for each 6-month period was calculated by counting as “exit” a herd not present in the next 6-month period and then classified as “Non-infection” or “Infection” depending on its last status. The “Recoveries” were identified by evaluating the change of status from infected in the previous 6-month period to susceptible in the following period.

A discrete-time negative binomial regression model (34) was implemented to estimate each of the 6-month herd-level indicators: entry rate (σ), exit rate unrelated to infection (μ_*j*_), exit rate related to infection (μ_*k*_), the recovery rate (r), and the transmission parameter (β). To estimate the transmission parameter (β), the following model was implemented:


$$\begin{array}{l} E\left[\ln\left(INC\right)\right]=\ln\left[\beta \right]+\ln\left[INS\;\times AP\;\right], \end{array}$$

where *E*[ln(INC)] is the expected log number of new infections per semester;

INS is the number of susceptible, calculated as N_t−1_–INF_t−1_, in each semester; and

AP is calculated as INF/*N*, where INF is the number of positive herds during the semester observed and *N* is the total number of tested herds in the same period.

The term ln[INS × AP] was used as an offset.

A negative binomial regression model was then implemented for each measure, by changing the input parameters:

- Entry rate (σ): using the number of entries (S.ENT + I.ENT) as response and the number of herds for each 6-month period as a model offset (ln[*N*]);
- Exit rate unrelated to infection (μ_j_): using the number of non-infection exits (NIE) as response and the initial number of susceptible as a model offset (ln[*INS*]), calculated as N_t−1_-INF_t−1_ ;
- Exit rate related to infection (μ_k_): using the number of infection exits (IE) as a response and the initial number of infected as a model offset (ln[*INI*]);
- Recovery rate (r): using the number of recoveries as a response (REC) and the initial number of infected as a model offset (ln[*INI*]).

The proportion of susceptible entries was then calculated as the ratio between the mean number of susceptible entries (S.ENT) and the mean of total entries in the period (S.ENT + I.ENT).

Finally, we used the parameter estimates to compute the basic reproduction number (R_0_) as:


$$\begin{array}{l} R_{0}=\beta /\left(r+\;\mu_{\text{k}}\right), \end{array}$$

where β is the transmission rate, r is the rate of infected herds becoming susceptible herds (recovery rate), and μ_k_ is the rate of infected herds exited from the population.

All statistical analyses were performed in R and RStudio, using the “tidyverse” and “MASS” packages (35, 36). The developed R script is available in the Supplementary Materials.

## Results

### Estimates of Prevalence, Incidence, and Transmission Parameter Among Dairy Herds

In total, 55 herds were excluded from the analysis because they had just one control in the period. A total of 17,056 BTM samples from 2,831 dairy herds were examined over the past 3 years, out of which 1,170 (6.8%) were positive for GBS and 236 (1.4%) were contaminated, requiring resampling.

The frequency distribution of the number of times the herds have been positive during the 3-year period is reported in Table 2. A total of 437 (15.4%) herds had been positive at least one time over the study period. Descriptive statistics on the entry, exit, recovery, incidence, and prevalence of GBS by semester are shown in Table 3.

**Table 2:** The frequency distribution of the number of times herds resulted positive for *Streptococcus agalactiae* during the period 2019–2021 (six semesters) in the Emilia-Romagna region.

**No. times infected**	**Frequency**	**Freq. %**
0	2,394	84.6
1	153	5.4
2	90	3.2
3	68	2.4
4	58	2.0
5	41	1.4
6	27	1.0
Total	2,831	100

**Table 3:** Descriptive statistics by semester of entry, exit, recovery, incidence and prevalence of *S. agalactiae* in the dairy herds of Emilia-Romagna region, 2019–2021.

**Year/semester**	***N***	**Entry**		**Infection exit and recovery**						**Non-infection exit and incidence**						**Prevalence**	
		**S.ENT**	**I.ENT**	**INI**	**ANI**	**IE**	**IEr %**	**REC**	**RECr %**	**INS**	**ANS**	**NIE**	**NIEr %**	**INC**	**INCr %**	**INF**	**AP %**
2019/1	2,721	NA	NA	NA	NA	NA	NA	NA	NA	NA	NA	16	NA	NA	NA	243	8.9%
2019/2	2,788	74	7	243	216.5	14	6.5%	118	54.5%	2,478	2,385.0	306	12.8%	72	3.0%	201	7.2%
2020/1	2,496	27	1	201	197.5	4	2.0%	76	38.5%	2,587	2,579.5	46	1.8%	72	2.8%	192	7.7%
2020/2	2,709	249	16	192	180.5	7	3.9%	85	47.1%	2,304	2,402.0	85	3.5%	53	2.2%	170	6.3%
2021/1	2,645	26	3	170	180.0	10	5.6%	56	31.1%	2,539	2,498.0	81	3.2%	83	3.3%	196	7.4%
2021/2	2,575	21	0	196	171.0	0	0.0%	87	50.9%	2,449	2,484.5	0	0.0%	37	1.5%	134	5.2%

*NA, not available; N, number of herds tested during the semester observed; S.ENT, susceptible entries, negative herds not present in the previous semester; I.ENT, infected entries, positive herds not present in the previous semester; INI, initial number of infected, number of herds infected at the previous semester; ANI, average number of infected, calculated as {INI + [0.5 × (I.ENT+INC-REC-IE)]}; IE, infected exits, positive herds not present in the next semester; IEr, infected exits rate, calculated as IE/ANI; REC, number of recoveries, changing status from I to NI from the previous semester; RECr, recovery rate, calculated as REC/ANI; INS, Initial number of susceptible, calculated as N_t−1_-INF_t−1_; ANS, average number of susceptible, calculated as {INS + [0.5 × (S.ENT+ REC-INC-NIE)]}; NIE, non-infection exits, number of positive herds not present in the next semester; NIEr, non-infection exit rate, calculated as NIE/ANS; INC, incidence count, count of new positive herds during the semester observed; INCr, incidence rate, calculated as INC/ANS; INF, number of infected, count of positive herds during the semester observed; AP, apparent prevalence, calculated as INF/N*.

**Figure 2:**
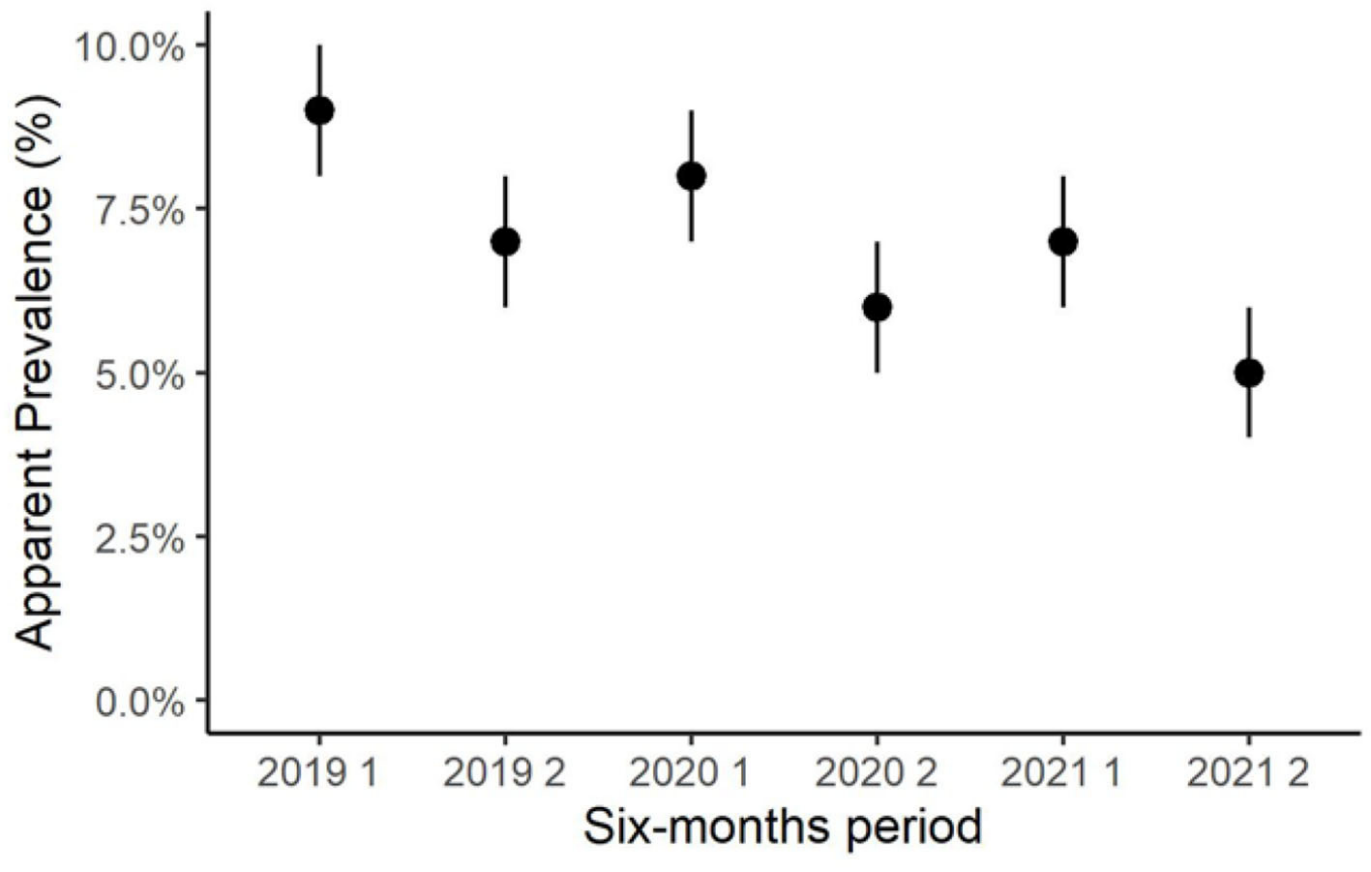
Trend of the semiannual herd-level apparent prevalence of *Streptococcus agalactiae* in dairy herds of the Emilia-Romagna region, 2019–2021.

**Figure 3:**
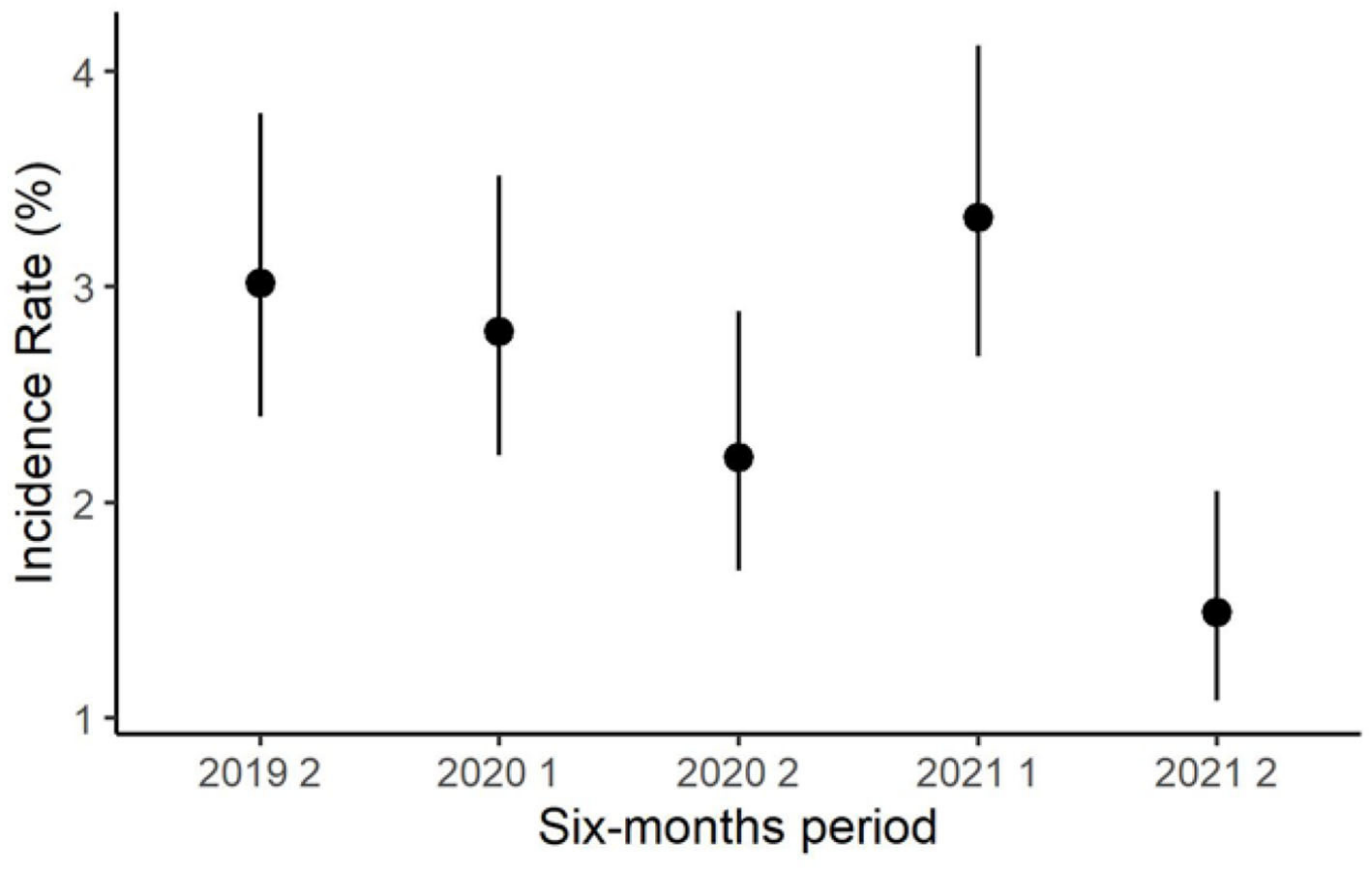
Trend of the semiannual herd-level incidence rate of *S. agalactiae* in dairy herds of the Emilia-Romagna region, 2019–2021.

During the observation period, the semiannual apparent prevalence decreased, from 8.9% in the first semester of 2019 to 5.2% in the second semester of 2021 (Figure 2). Notably, the prevalence resulted higher in the first half of each year than in the second half of the same year. In the first semester of 2019, the incidence rate was not computed because all positive herds were considered prevalent. The semiannual incidence rate decreased from 3.0% in the second semester of 2019 to 1.5% in the second semester of 2021 (Figure 3).

The herd-level parameter estimates per 100 herd-years at risk are shown in Table 4. In detail, the entry rate (6.3) was lower than the exit rate unrelated to the infection (8.4) but was not far from the exit rate related to the infection (6.9). The proportion of entries entered as susceptible is higher (0.93) compared with the proportion of herds entered as infected (0.07).

**Table 4:** Parameter estimates in a negative binomial regression model representing the dynamic of *S. agalactiae* transmission among the dairy herds of the Emilia-Romagna region.

**Parameter (symbol)**	**Estimate**	**95% confidence intervals**
Entry rate (σ)	6.3	3.1–15.9
Exit rate unrelated to infection (μ_j_)	8.4	2.9–43.7
Exit rate related to infection (μ_k_)	6.9	3.4–16.1
Recovery rate (r)	83.9	74.7–94.0
Transmission parameter (β)	124.3	115.7–132.4
Proportion of susceptible entries (ϑ)	93.6	
Reproductive number (R_0_)	1.4	

*Rates of entry, exit, recovery, and transmission are expressed as events per 100 herd-years at risk*.

The transmission parameter β was estimated to be 124.3 new herd infections per 100 herd years at risk, and the reproductive number (*R*_0_) computed using the estimated parameters was equal to 1.4.

### SCCs in Dairy Herd

**Figure 4:**
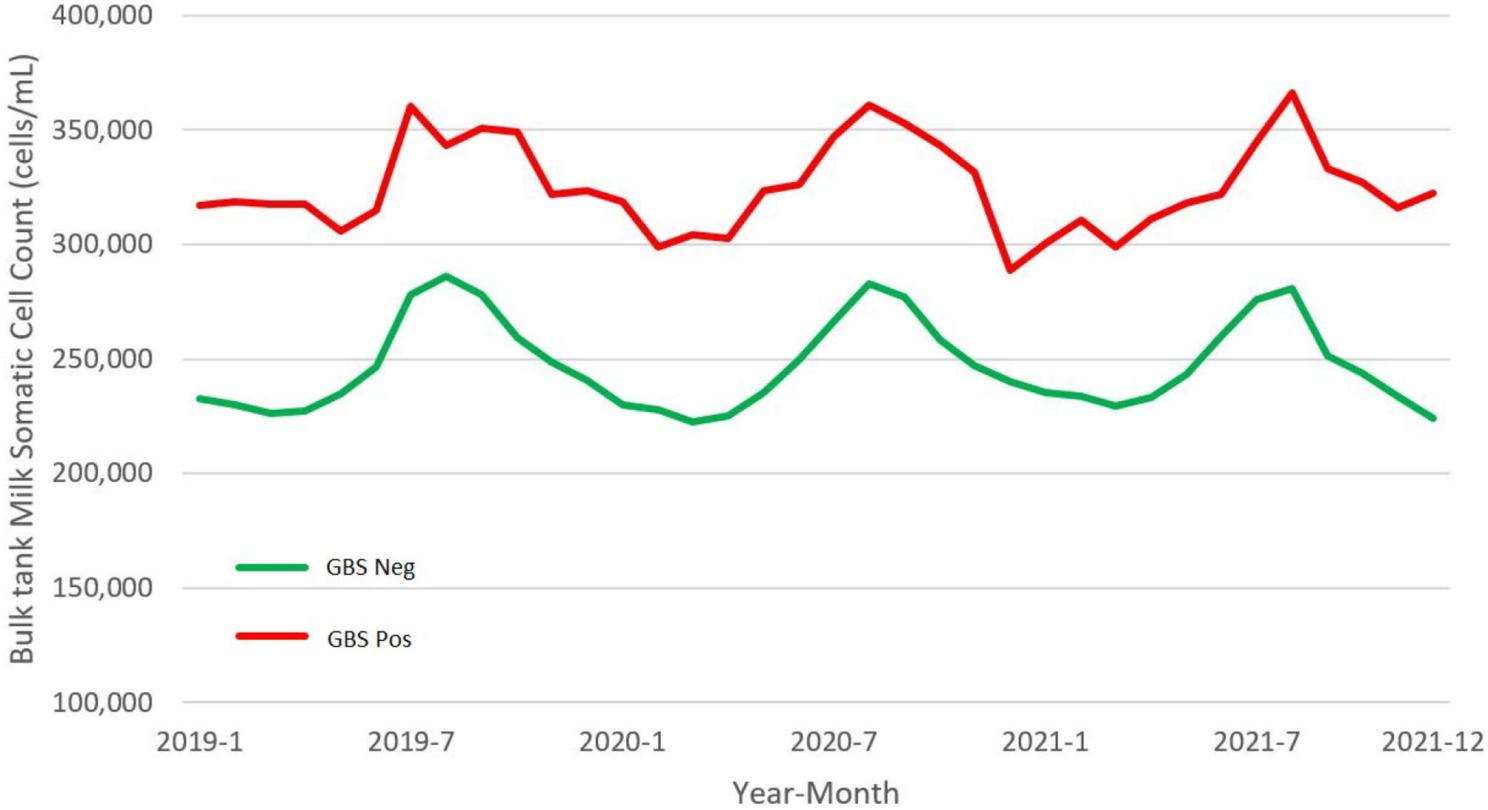
Monthly mean of bulk tank milk (BTM) somatic cell counts (SCCs) in dairy herds positive and negative for *S. agalactiae* (GBS) of the Emilia-Romagna region, 2019–2021.

On average for each semester, we collected the data about BTM SCC from 97.3% (range: 96.8–98.3%) of the herds tested for GBS. Positive herds constantly showed a BTM SCC higher than negative ones (Figure 4), and on average, the difference scored about 77,000 cells/ml (GBS positive farms: 324,526 ± 169,387 cells/ml; GBS negative: 247,660 ± 142,452 cells/ml). In Italy, to be marketed, milk shall have an SCC not exceeding 400,000 cells/ml, expressed as the geometric mean of the SCC recorded in a 3-month period, with at least one sample per month. In Table 5, we report the number of herds that, during the observational period, exceeded this threshold. On average, GBS positive herds had a 3.2 (95% *CI*s: 2.1–4.9) higher risk of producing milk non-compliant with SCC permissible threshold.

**Table 5:** The number of dairy herds with a bulk tank milk (BTM) somatic cell counts (SCCs) exceeding the legal limit, by *S. agalactiae* status and semester, in Emilia-Romagna region, 2019–2021.

	***Streptococcus agalactiae*** **positive**						***Streptococcus agalactiae*** **Negative**						
	**Herds**			**Samplings for SCC**			**Herds**			**Samplings for SCC**			
**Year/ semester**	**Sampled**	Non-compliant*	**NC herds %**	**Total samplings**	Non-compliant*	**NC samplings %**	**Sampled**	Non-compliant*	**NC herds %**	**Total samplings**	Non-compliant*	**NC samplings %**	**Herd prevalence ratio**
2019/1	236	32	13.6%	541	59	10.9%	2,440	115	4.7%	5,847	214	3.7%	2.9
2019/2	192	27	14.1%	473	56	11.8%	2,508	93	3.7%	5,971	183	3.1%	3.8
2020/1	187	26	13.9%	428	60	14.0%	2,228	94	4.2%	5,266	164	3.1%	3.3
2020/2	164	26	15.9%	380	55	14.5%	2,458	86	3.5%	5,791	172	3.0%	4.5
2021/1	189	22	11.6%	460	37	8.0%	2,392	100	4.2%	6,057	235	3.9%	2.8
2021/2	128	10	7.8%	294	18	6.1%	2,385	96	4.0%	6,012	206	3.4%	1.9
Mean	183	24	13.0%	429	48	11.1%	2,402	97	4.1%	5,824	196	3.4%	3.2

*Non-compliant (NC): bulk tank milk somatic cell count moving average >400,000 cells/ml.

## Discussion

In the first 3 years of the program in the Emilia-Romagna region, the apparent herd prevalence of GBS decreased from 8.9 to 5.2%. These values are comparable to those recently recorded in the Lombardy region, which is the only other Italian region carrying out a control program against this mastitis agent (30, 31). Prevalence and incidence values are higher in these case regions than in Northern European countries, such as Denmark, where GBS eradication programs have been in place for many years (16). However, some recent findings in Northern European countries support the re-emergence of GBS in the dairy population due to human-to-cattle transmission and demonstrate that reverse zoonotic transmission can erase the successes of animal disease control campaigns (18, 27).

Despite a slight decreasing trend of both prevalence and incidence, the reproductive number R_0_ was estimated at 1.4, indicating an active spread of GBS in the dairy cattle population of the Emilia-Romagna region. This apparent discrepancy could be partially due to an overestimation of the transmission rate β in our model, due to some factors influencing the incidence rate calculation, such as false-negative results of the BTM test and missing values related to uncontrolled herds. The bacteriological culture on BTM has a low sensitivity, not only related to the intrinsic characteristics of the diagnostic procedure but also to the fact that GBS infected dry cows and cows treated with antimicrobials or presenting clinical mastitis did not contribute to the BTM (5, 37). After every BTM culture positive result for GBS, the Emilia-Romagna regional control program requires the farmer to implement the following actions: (i) test the culture of composite individual milk samples from each lactating cow; (ii) treat every positive cow with antibiotics; and (iii) cull cows with chronic infection. However, after this first line of control actions, the adoption of a continuing herd eradication program is voluntary and only a few farmers have undertaken it since the beginning of the GBS control plan in 2019. It is possible, however, that this sporadic intervention has lowered the within-herd prevalence enough to give a false negative result to the following BTM sampling. In the six semesters considered within the framework of this study, the number of farms with a series of positive-negative-positive results were 136/437 (31.1%) and were all considered as reinfections, but probably only a part of these had been recovered at the moment of the negative result. Since uncontrolled herds were considered to have left the population and as new entries at the next sampling within the model, it is possible that this assumption slightly biased the estimation of the transmission parameters entering into the calculation of R_0_. Unfortunately, we had several missing values, especially in the first half of 2020, when restrictions on people's movements imposed to counteract the COVID-19 pandemic limited both sampling and laboratory activities.

However, our data show that the transmission of GBS among farms in the Emilia-Romagna region occurred, and it was probably facilitated by the absence of restrictions on animal movements for infected farms. The purchase of animals from infected herds is one of the most important risk factor for introducing GBS in uninfected herds (19, 20). In the Emilia-Romagna region, as in most European countries, there has been a decrease in the number of farms, but an increase in their size (Table 1) (16, 31). Hence, even if several farms exited from the population, most of the animals remained in it, increasing the risk of GBS spreading, because they were purchased by other farmers.

The role of personnel as a possible source of infection should not be excluded (17, 18), since infections with strains shared between humans and cattle have been identified in several studies (26, 28). Finally, as a possible cause of reinfection, the role of “environmental” GBS strains, such as ST 103, which can colonize the intestinal tract of cattle and contaminate barns and drinking troughs, should be considered (11). Recently, this strain has been reported also in Italy (12, 26).

The BTM SCC is inversely related to the quantity and quality of milk produced (8, 38). Farms infected with GBS in the Emilia-Romagna region have a consistently higher SCC than negative ones. On average, this difference was estimated to be 77,000 cells/ml, corresponding to a milk loss of 0.4 kg/cow/day, using the equation proposed by Eberhart et al. (38). GBS infected farms also have a three times higher risk than the negative ones of having non-marketable milk by exceeding the SCC limit.

These figures indicate that the eradication of GBS from the dairy herds of the Emilia-Romagna region is economically viable and that the control program should be maintained, increasing the awareness of farmers that GBS significantly limits the profitability of their herds.

## Data Availability Statement

The raw data supporting the conclusions of this article will be made available by the authors, without undue reservation.

## Author Contributions

MT, EM, and NA took part in designing the survey. NA, AP, GP, and PB tested the bulk milk samples. MT, RR, GG, AS, and GC performed data collection, data analysis, and wrote the paper. All authors participated in reading, provided a critical review, and approved the final manuscript.

## Funding

This work is part of the SOUND-control project (https://sound-control.eu/), funded by the Horizon 2020 Framework Programme of the European Union and COST-European Cooperation in Science and Technology (Cost Action CA17110).

## Conflict of Interest

The authors declare that the research was conducted in the absence of any commercial or financial relationships that could be construed as a potential conflict of interest.

## Publisher's Note

All claims expressed in this article are solely those of the authors and do not necessarily represent those of their affiliated organizations, or those of the publisher, the editors and the reviewers. Any product that may be evaluated in this article, or claim that may be made by its manufacturer, is not guaranteed or endorsed by the publisher.
